# Supplementary figures and images for: Experimental study on the various varieties of photovoltaic panels (PVs) cooling systems to increase their electrical efficiency
Source: PLoS One. 2024 Sep 16;19(9):e0307616. doi: 10.1371/journal.pone.0307616 (PMC11404811; doi:10.1371/journal.pone.0307616)

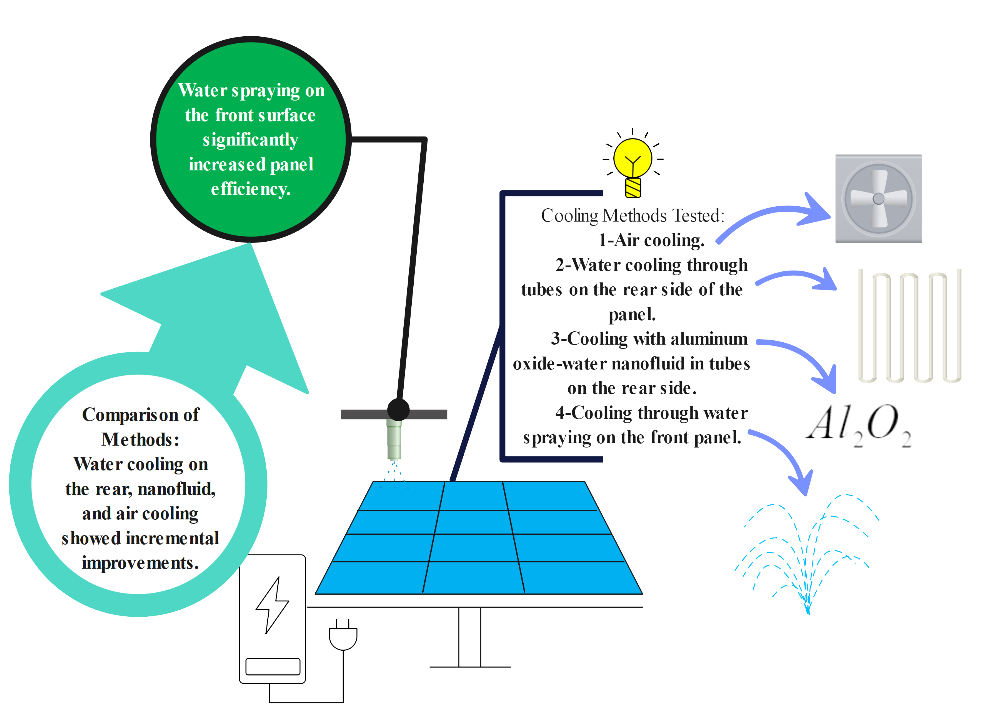

Supplement: S1 Graphical abstract — (TIF) [file pone.0307616.s001.tif]
